# SBI and EHR: understanding, adoption, and implementation in family medicine clinics

**DOI:** 10.1186/1940-0640-10-S2-O48

**Published:** 2015-09-24

**Authors:** Roger Zoorob, Sandra J Gonzalez, Heather Snell, Heather O'Hara, Mohamad Sidani

**Affiliations:** 1 Family and Community Medicine, Baylor College of Medicine, Houston, USA; 2 Family and Community Medicine, Meharry Medical College, Nashville, USA

## Background

Alcohol screening and brief intervention (SBI) programs have been shown to be effective in reducing risky alcohol consumption among primary care patients [1-3]. Although various implementation protocols exist, it can be difficult to launch and sustain SBI programs. A number of barriers exist, including those related to clinical workflow, the intake process, and the incorporation of protocols into electronic health records (EHR)[4-6].

This study aims to present challenges and potential solutions to incorporating SBI as a standard of care into an existing EHR of a family medicine system.

## Material and methods

An SBI program was piloted in two underserved family medicine clinical teaching practices. Physicians, residents, nurses, medical assistants and patient service representatives (PSR's) were trained on the protocol for adopting this practice into daily clinic work. Through this implementation, an EHR template was created to complement the workflow.

## Results

Utilization of the screening tool provided a mechanism to better assess risky drinking within a regular patient encounter. High rates of completion were appreciated throughout the grant period prior to leadership changes. The EHR template facilitated the intake process, the clinical encounter, and provided a mechanism for billing.

## Conclusions

The incorporation of a dedicated EHR template may mitigate providers’ concerns about time constraints and establishes a more effective mechanism for billing for the service.

Strong support from organizational leadership and the use of clinic champions were shown to positively contribute to the success of the SBI programs in this health system by addressing common barriers to implementation.
